# Molecular and Genetics Perspectives on Primary Adrenocortical Hyperfunction Disorders

**DOI:** 10.3390/ijms252111341

**Published:** 2024-10-22

**Authors:** Sanggu Kim, Preeti Kumari Chaudhary, Soochong Kim

**Affiliations:** College of Veterinary Medicine, Chungbuk National University, Cheongju 28644, Republic of Korea; tkdrnfld@naver.com (S.K.); chaudharypreety11@gmail.com (P.K.C.)

**Keywords:** adrenal gland, primary adrenocortical hyperfunction, adrenal hyperplasia, primary aldosteronism, carcinoma

## Abstract

Adrenocortical disorders encompass a broad spectrum of conditions ranging from benign hyperplasia to malignant tumors, significantly disrupting hormone balance and causing a variety of clinical manifestations. By leveraging next-generation sequencing and in silico analyses, recent studies have uncovered the genetic and molecular pathways implicated in these transitions. In this review, we explored the molecular and genetic alterations in adrenocortical disorders, with a particular focus on the transitions from normal adrenal function to hyperfunction. The insights gained are intended to enhance diagnostic and therapeutic strategies, offering up-to-date knowledge for managing these complex conditions effectively.

## 1. Introduction

The adrenal gland, a key organ located in the retroperitoneum, plays an essential role in maintaining homeostasis and orchestrating the body’s response to endocrine stress [1,2]. This gland consists of two distinct parts: the cortex and medulla, each vital for producing critical hormones. Within the adrenal cortex, there are three zones: the zona glomerulosa (ZG), zona fasciculata (ZF), and zona reticularis (ZR), which predominantly generate aldosterone (mineralocorticoids), cortisol (glucocorticoids), and androgen, respectively. These hormones are crucial for a myriad of physiological processes, including growth, metabolism, reproduction, immune responses, and stress management [3,4].

However, adrenocortical disorders encompass a broad spectrum of conditions ranging from benign hyperplasia to malignant tumors that can significantly disrupt the balance of steroid hormones, leading to a spectrum of pathophysiological manifestations. Adrenocortical disorders are broadly classified as adrenal insufficiency (AI), characterized by insufficient hormone production, and conditions related to adrenocortical hyperfunction, which leads to excessive hormone production. Although rare, with AI presenting in 0.01–0.02% of the population and overactive adrenal conditions occurring even less frequently at rates of 0.0001–0.0003%, the clinical implications are significant. AI has a mortality rate of approximately 15%, whereas disorders associated with adrenal overactivity have a five-year survival rate of only 50%, underscoring the critical need for effective management and a deeper understanding [5]. For example, the overproduction of cortisol in Cushing’s syndrome and aldosterone in Conn’s syndrome illustrate how hyper functional states can contribute to significant morbidity.

Recent molecular biology studies, particularly using next-generation sequencing and in silico analyses, have identified genetic and molecular pathways implicated in the pathogenesis of adrenocortical disorders. The primary aim of this review is to provide a comprehensive overview of the molecular and genetic alterations involved in primary adrenocortical hyperfunction disorders, with a particular focus on the transition from normal adrenal physiology to hyperfunctional states. By exploring the latest advancements in next-generation sequencing and in silico analyses, we aim to uncover the underlying molecular mechanisms driving these conditions and discuss their implications for improving diagnostic and therapeutic strategies. The insights presented here are intended to bridge the gap between clinical manifestations and molecular biology, offering up-to-date knowledge for clinicians and researchers working to manage these complex disorders effectively.

## 2. Normal Physiology of the Adrenal Cortex

The adrenal cortex is divided into three histomorphological, functional, and anatomically distinct zones (ZG, ZF, and ZR). The primary role of the adrenal cortex is to generate adrenal steroid hormones, which are produced in all three cell zones [2]. Adrenal steroidogenesis begins with converting lipoproteins into cholesterol by activating adrenocortical cells via various signaling pathways [6]. Subsequently, adrenocorticotropic hormone (ACTH) increases the expression of steroidogenic acute regulatory proteins, and multiple signaling mechanisms allow cholesterol to reach the mitochondrial membrane [7,8]. Cholesterol is further converted to pregnenolone by CYP11A or cholesterol desmolase and released into the cytoplasm, where it is tightly regulated by various processes, including paracrine and endocrine reactions, depending on the type of steroid produced by adrenal cortex cells [9]. These adrenal cortical hormones bind to certain nuclear steroid receptors and perform various physiological functions. Glucocorticoids bind to the glucocorticoid receptor (GR), minerals to the mineralocorticoid receptor (MR), and androgens to the androgen receptor (AR) [10,11].

### 2.1. Zones of Adrenal Cortex

#### 2.1.1. Zona Glomerulosa

The ZG, the outermost component of the adrenal cortex, accounts for approximately 15% of the cortex and is mainly controlled by the renin-angiotensin system, potassium, and ACTH, with minor contributions from dopamine, atrial natriuretic peptide, and various neuropeptides [12]. In ZG, pregnenolone is transformed to progesterone by 3β-hydroxysteroid dehydrogenase (3β-HSD), which is then converted to deoxycorticosterone by 21-hydroxylation by CYP21 or 21-hydroxylase. Deoxycorticosterone undergoes 11β-hydroxylation to produce corticosterone, which is then converted to aldosterone by 18-hydroxylation and 18-oxidation [13]. Through these reactions, ZG produces aldosterone, a mineralocorticoid that regulates blood pressure by managing electrolyte balance via salt and water retention and intravascular volume [2,14,15].

#### 2.1.2. Zona Fasciculata

The ZF lies beneath the ZG and is a key primary site of adrenal gland glucocorticoid production, which is directly regulated by pituitary corticotroph cells secreting ACTH. Furthermore, numerous cytokines, including interleukin-1, interleukin-6, tumor necrosis factor, neuropeptides, and catecholamines, affect glucocorticoid production. The ZF contains large, lipid-rich cells that produce and release cortisol [16]. ACTH in the ZF causes pregnenolone to be converted to 17α-hydroxypregnenolone in the endoplasmic reticulum by the enzyme CYP17 or 17α-hydroxylase/17,20-lyase (P450c17) and then further converted to 17α-hydroxyprogesterone, 21-hydroxylated, and 11-deoxycortisol by 3β-HSD and CYP21 [17]. Finally, the enzyme CYP11B1 or 11β-hydroxylase converts 11-deoxycortisol to cortisol [16]. This hormone has a significant influence on the immunological, metabolic, and cardiovascular systems.

#### 2.1.3. Zona Reticularis

The ZR is the inner zone and is responsible for the production of certain glucocorticoids (cortisol and corticosterone), as well as adrenal androgens such as Δ4-androstenedione, dehydroepiandrosterone (DHEA), and dehydroepiandrosterone sulfate (DHEAS), which are directly regulated by the secretion of ACTH [2,18]. The ZR is composed of cells with lipofuscin granules, which are responsible for adrenal androgen production and secretion. Pregnenolone undergoes 17α-hydroxylation in the ZR to become 17α-hydroxypregnenolone, which is then further transformed into DHEA by the enzyme CYP17 [2]. Additionally, 3β-HSD converts DHEA to Δ4-androstenedione, and SULT2A1 converts it to DHEAS. Finally, in the gonads, Δ4-androstenedione is transformed into testosterone by 17β-hydroxysteroid dehydrogenase in males and into estrone and 17β-estradiol by CYP19 or aromatase in females [16,19,20,21,22,23]. These hormones exert significant effects, particularly during puberty [24].

### 2.2. Hypothalamus–Pituitary–Adrenal Axis and Steroidogenesis

**Figure 1:**
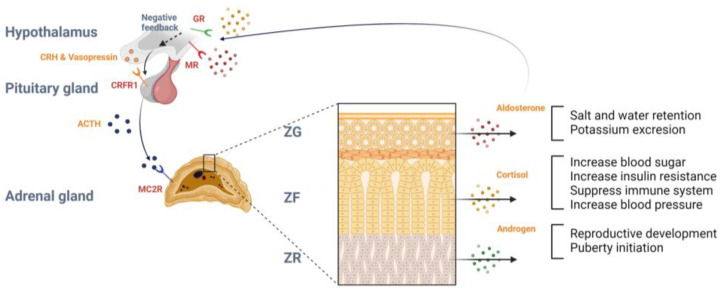
Schematic overview of the HPA axis and function of the adrenal cortex. Abbreviations: GR, glucocorticoid receptor; MR, mineralocorticoid receptor; CRH, corticotropin-releasing hormone; CRFR1, corticotropin-releasing factor type 1 receptor; MC2R, melanocortin type 2 receptor; ZG, zona glomerulosa; ZF, zona fasciculata; ZR, zona reticularis.

The hypothalamus–pituitary–adrenal (HPA) axis controls and regulates the adrenal hormonal system (Figure 1). According to the circadian rhythm, the HPA axis is physiologically active in the morning and is mostly activated under stressful or threatened conditions to maintain normal physiological homeostasis [25,26]. Moreover, it biosynthesizes corticotropin-releasing hormone (CRH) and vasopressin in the hypothalamus paraventricular nucleus. When the HPA axis is initiated, CRH and vasopressin are secreted from the hypothalamus to the hypophysial portal vessel and transported to the anterior pituitary gland [27,28]. The pituitary gland precursor protein pro-opiomelanocortin is cleaved into ACTH by enzyme proprotein convertases 1 and 3 [29,30]. CRH binds to the corticotropin-releasing factor type 1 receptor of the anterior pituitary gland, activating the cyclic adenosine monophosphate (cAMP) pathway and releasing ACTH into the bloodstream, which is enhanced by vasopressin. The released ACTH binds to the melanocortin type 2 receptor (MC2R) in each adrenocortical gland zone, resulting in the production and secretion of glucocorticoids, mineralocorticoids, and androgens [31,32,33]. Secreted glucocorticoids and mineralocorticoids lead to negative feedback in the HPA axis by binding to the GR and MR in the paraventricular nucleus and hippocampus [34].

### 2.3. Functional Role of Steroid Hormones

Compared to adrenomedullary catecholamines, adrenocortical steroid hormones act more slowly but last for several hours. These steroid hormones affect a wide range of physiological processes by binding to the GRs, MRs, ARs, estrogen receptors (ERs), and progesterone receptors in various target cells.

Glucocorticoids function as both genomic and nongenomic managers by binding to the GRs. Genomic GR signaling promotes gene expression by directly binding to DNA (direct), tethering to other DNA-bound transcription factors (tethering), or interacting with neighboring DNA-bound transcription factors (composite) following direct DNA binding [35]. Furthermore, nongenomic GR signaling occurs when binding to cytosolic or membrane GR and occurs quickly owing to the absence of transcription and translation [36]. They function by activating several kinases, including mitogen-activated protein kinases (MAPKs), phosphoinositide 3-kinase, and AKT [37]. GR activation causes several physiological effects, such as apoptosis, stimulation, and differentiation of osteoblasts, osteoclasts, endothelial cells, smooth muscles, hepatocytes, pancreatic beta cells, and numerous immune cells [38,39,40,41].

Mineralocorticoids function as genomic or nongenomic managers by binding to MRs, similarly to GR [42]. Aldosterone, a representative mineralocorticoid, is mainly released by angiotensin II and directly activates the apical sodium transporter and sodium/potassium pump, which regulate the salt and water balance via sodium reabsorption [43]. They primarily affect the gut, kidneys, and salivary glands to promote passive water reabsorption, which regulates blood pressure [44].

Adrenocational androgens, such as DHEA and DHEAS, have a lower binding capacity than other sex steroid hormones, such as testosterone and estradiol, but act directly on AR and ER [45]. Androgens are the main hormones responsible for fetal development and puberty in young children [24]. Additionally, it functions as an antagonist of cortisol, an antidepressant, and causes irregular menstruation and related symptoms in women.

## 3. Adrenocortical Hyperfunction

Hyperfunction of the adrenocortical gland, resulting from various etiologies, including adrenal hyperplasia, adenomas, and carcinomas, primarily manifests as a spectrum of disorders characterized by excessive hormone production. These disorders, including hypercortisolism (also known as Cushing’s syndrome), aldosteronism, and hyperandrogenism, represent significant challenges in endocrinology owing to their complex clinical presentations and severe metabolic disruptions.

**Figure 2:**
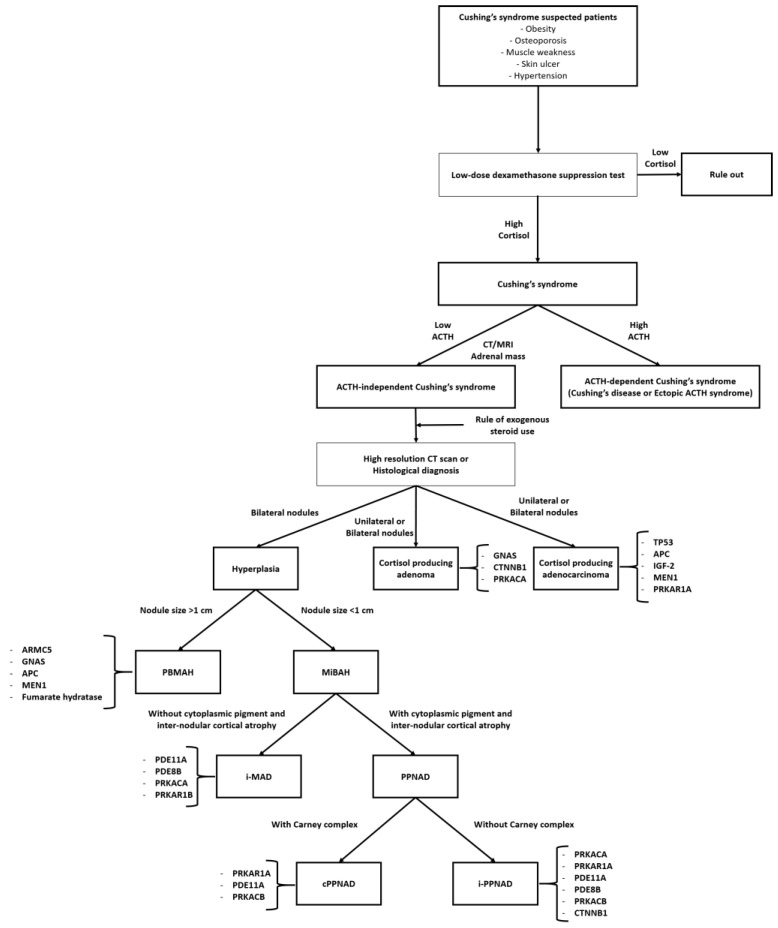
Diagnostic flow of Cushing’s syndrome and related genetic mutations. Abbreviations: PBMAH, primary bilateral macronodular adrenal hyperplasia; MiBAH, micronodular bilateral adrenal hyperplasia; i-MAD, isolated micronodular adrenocortical disease; PPNAD, primary pigmented micronodular adrenocortical disease; cPPNAD, Carney complex primary pigmented micronodular adrenocortical disease; i-PPNAD, isolated primary pigmented micronodular adrenocortical disease; *GNAS*, guanine nucleotide-binding protein alpha stimulating activity polypeptide; *CTNNB1*, catenin beta 1; *PRKACA*, protein kinase A catalytic subunit α; *TP53*, tumor protein p53; *APC*, adenomatous polyposis coli; *IGF-2*, insulin-like growth factor 2; *MEN1*, multiple endocrine neoplasia type 1; *PRKAR1A*, protein kinase A regulatory subunit α, *ARMC5,* armadillo repeat containing 5; *FH*, fumarate hydratase; *PDE11A*, phosphodiesterase 11A; *PDE8B*, phosphodiesterase 8B; *PRKAR1B*, protein kinase A regulatory subunit β; *PRKACB*, protein kinase A catalytic subunit β.

**Figure 3:**
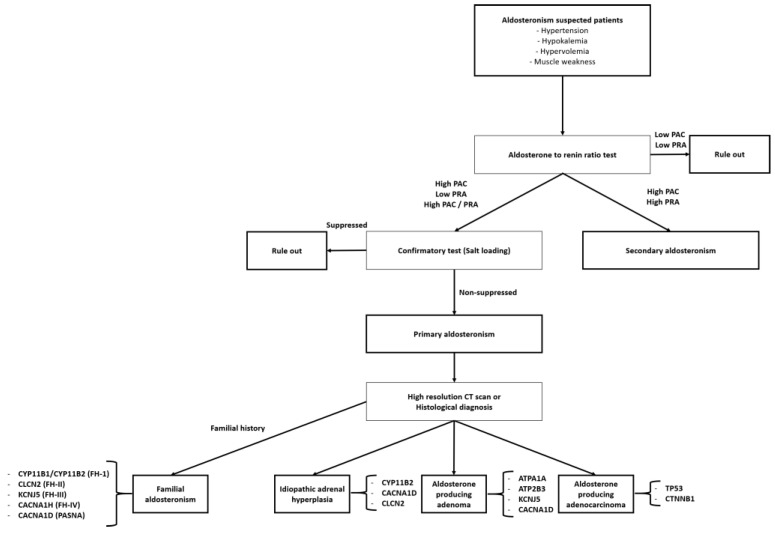
The diagnostic flow of aldosteronism and related genetic mutation. Abbreviations: PAC, plasma aldosterone concentration; PRA, plasma renin activity; *CYP11B1*, cytochrome P450 family 11 subfamily B member 1; *CYP11B2*, cytochrome P450 family 11 subfamily B member 2; *CLCN2*, chloride voltage-gated channel 2; *KCNJ5*, potassium inwardly-rectifying channel subfamily J member 5; *CACNA1H*, calcium voltage-gated channel subunit α1 H; *CACNA1D*, calcium voltage-gated channel subunit α1 D; *ATPA1A*, ATPase Na^+^/K^+^ transporting subunit α1; *ATP2B3*, ATPase plasma membrane Ca^2+^ transporting 3; *TP53*, tumor protein p53; *CTNNB1*, catenin beta 1.

Hypercortisolism can occur when large amounts of glucocorticoids are secreted and can be further classified as ACTH-independent Cushing’s syndrome (adrenal tumors), Cushing’s disease (pituitary tumors), or ectopic ACTH syndrome based on the etiology (Figure 2). Excessive aldosterone secretion is referred to as aldosteronism and is classified as primary (Conn’s syndrome with low renin/angiotensin II) or secondary (high renin/angiotensin II) (Figure 3). Hyperandrogenism is characterized by elevated levels of androgenic hormones. Because chronic oversecretion of adrenal hormones can induce substantial side effects, even at minimal levels, an understanding of the underlying molecular mechanisms and proper treatment is required.

Adrenocortical hyperfunction can cause a variety of immunological dysregulations. Hypercortisolism stimulates the immune system through its potent anti-inflammatory and immunosuppressive properties [46]. Furthermore, dysregulated immune responses alter cytokine production, increasing the risk of infection and inhibiting immune tumor regression [47]. Aldosteronism stimulates macrophages and T lymphocytes, which contributes to mild inflammation [48]. Furthermore, excess aldosterone can induce oxidative stress, which activates proinflammatory pathways in immune cells. This can lead to endothelial damage, vascular remodeling, and fibrosis, particularly in the kidneys, heart, and blood vessels. Hyperandrogenism can also cause moderate inflammation and elevate inflammatory markers such as C-reactive protein, interleukin-6, and TNF-α [49]. Moreover, androgens induce a proinflammatory M1 macrophage phenotype and increase T-cell cytokine production. Since such immunodysregulation can result in a number of adverse effects and autoimmune disorders due to chronic inflammation, appropriate therapy is crucial.

**Figure 4:**
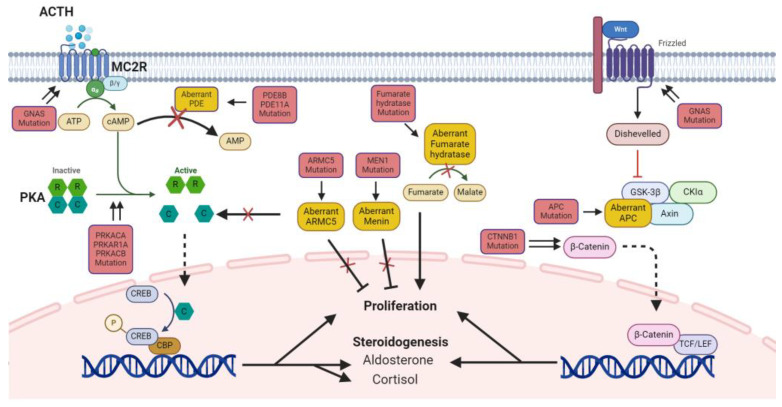
Diagram of mutations and signaling alterations in adrenal hyperfunction. Red boxes indicate gene mutation and yellow boxes indicate aberrant molecules. Double arrows mean activating mutations and arrows with X mean inhibited signaling by aberrant molecules. Dashed arrows (---→) indicate an anticipated outcome; red solid lines indicate activating mutations, showing how mutations affect downstream pathways; black solid lines with X show inhibited signaling caused by aberrant molecules or mutations, indicating that the normal pathway is blocked. Abbreviations: ACTH, adrenocorticotropic hormone; MC2R, melanocortin type 2 receptor; *GNAS*, guanine nucleotide-binding protein alpha stimulating activity polypeptide; cAMP, cyclic adenosine monophosphate; PDE, phosphodiesterase; PKA, protein kinase A; *PRKACA*, protein kinase A catalytic subunit α; *PRKAR1A*, protein kinase A regulatory subunit α; *PRKACB*, protein kinase A catalytic subunit β; *ARMC5*, armadillo repeat containing 5; *MEN1*, multiple endocrine neoplasia type 1; *CTNNB1*, catenin beta 1; *APC*, adenomatous polyposis coli; CREB, cAMP-response element binding protein; CBP, CREB-binding protein.

Adrenocortical hyperfunction can be classified as primary or ACTH-independent (adrenocortical tumors) or secondary or ACTH-dependent (excessive adrenal stimulating factors). Adrenocortical hyperplasia, adenomas, and carcinomas are common and they affect 4–7% of people over the age of 40 and 5–10% of those over the age of 70 years [50]. Approximately 55% of these are functional, causing an increased secretion of glucocorticoids, mineralocorticoids, and androgens, either separately or concurrently [51]. Cushing’s syndrome occurs in >75% of patients with adrenal adenomas, 10% of patients with adrenal hyperplasia, and 5% of patients with adrenal adenocarcinoma [52]. Patients with adrenocortical tumors have an overall 5-year survival rate of 40–60%, indicating poor prognosis [53,54]. To help understand adrenocortical tumors and achieve a better prognosis, we discuss the latest molecular and genetic mechanisms of primary adrenocortical hyperfunction (Figure 4).

### 3.1. Adrenal Hyperplasia

#### 3.1.1. Primary Bilateral Macronodular Adrenocortical Hyperplasia

Approximately 10% of Cushing’s syndrome cases are caused by adrenocortical hyperplasia, and less than 2% of Cushing’s syndrome cases are caused by primary bilateral macronodular adrenal hyperplasia (PBMAH), which is characterized by benign macronodules larger than 1 cm [55]. Most PBMAHs are diagnosed in patients aged 40–65 years. However, because PBMAH is characterized by multiple genetic predispositions, it was proposed to be referred to as a primary bilateral macronodular adrenocortical disease in the 2022 World Health Organization pathological classification [56]. PBMAH was previously considered ACTH-independent; however, the paracrine effects of intra-adrenal ACTH secretion appeared in a subset of steroidogenic cells in hyperplastic adrenal glands, suggesting that it may be ACTH-dependent [57]. The most prevalent is hypercortisolism, the rarest is hyperandrogenism, and over 90% of hypercortisolism cases are functional but mild [58].

Next-generation sequencing analysis identified PBMAH as a tumor resulting from several germline and somatic mutations. The most notable mutation is the armadillo repeat containing 5 (*ARMC5*) gene mutation, which has been shown in 15–26% of patients with PBMAH and is known to disrupt the G1 phase and cell cycle and cause cell death [58,59,60,61]. Inhibition of the *ARMC5* gene increases tumorigenesis, reduces apoptosis, and prevents its interaction with Cullin3, which degrades ARMC5, further inhibiting ARMC5 degradation [62]. Paradoxically, inhibition of *ARMC5* leads to MC2R expression inhibition, *CYP17A1* and *CYP21A2* mRNA reduction, cAMP-response element binding protein (CREB) signaling inhibition through protein kinase A (PKA) inhibition, redox homeostasis disruption through ubiquitination with nuclear respiratory factor 1, and decreased sterol regulatory element-binding transcription factor activity, which inhibits steroidogenesis and cell proliferation. However, increased absolute steroidogenesis by Wnt-signaling-induced malignant tumor development due to PKA activity inhibition and large adrenal mass plays a more significant role [60,63,64,65,66]. Inactivation of ARMC5 leads to aberrant expression of G-protein-coupled receptors, such as β-adrenergic, α2-adrenergic, and V1-vasopressin receptors, which promote steroidogenesis [67,68]. Furthermore, a recent study revealed that an *ARMC5*-inactivating mutation decreases sirtuin 1 activity, which may promote cortisol hypersecretion via aberrant protein acetylation [69].

In most cases, PBMAH caused by *ARMC5* mutation results in hypercortisolism; however, in rare situations, hyperaldosteronism occurs [70]. Nevertheless, recent studies have revealed that steroidogenesis can be inhibited in patients with *ARMC5* mutations, especially because androgen synthesis is particularly dysregulated; however, further studies are required [71].

Furthermore, mutations in the lysine (K)-specific demethylase 1A (*KDM1A*) and glucose-dependent insulinotropic polypeptide receptor (*GIPR*) genes result in GIP-dependent PBMAH. Inactivation of *KDM1A* and duplication of the *GIPR* gene result in the overexpression of GIPR and the overexpression or downregulation of other G-protein-coupled receptors. Food ingestion promotes GIP signaling in the adrenal cortex, which induces cortisol secretion in patients with *KDM1A* and *GIPR* mutations [72]. These two mutations upregulate the luteinizing hormone (LH)/choriogonadotropin receptor, suggesting they may be related to hypercortisolism associated with pregnancy and menopause [73]. Additionally, several gene mutations, including *fumarate hydratase* (cAMP and PKA pathway inhibition), *MEN1* (menin-induced inhibition of several tumor suppressor pathways), *APC* (Wnt/β-catenin signaling stimulation, GIP, 5-HT7, and abnormal LH receptor expression), *GNAS* (GNAS/PKA/CREB activation), *EDNRA* (MAPK signaling activation), L205R in *PRKACA* (PKA pathway activation), *MC2R* (MC2R/PKA/CREB activation), and *PDE8B* and *PDE11A* (cAMP hydrolysis and PKA pathway inhibition), inhibit tumor suppression and enhance steroidogenesis [74,75,76,77,78,79,80,81,82,83]. According to the latest bioinformatics analysis, the downregulation of *GPC4* and *VCAN* may also be involved in PBMAH development [84].

#### 3.1.2. Micronodular Bilateral Adrenal Hyperplasia

Unlike PBMAH, micronodular bilateral adrenal hyperplasia is defined as nodular hyperplasia with a diameter of less than 1 cm and is usually diagnosed in children and young adults. Micronodular bilateral adrenal hyperplasia is generally classified into two types: isolated micronodular adrenocortical disease and primary pigmented micronodular adrenocortical disease (PPNAD), which are PPNAD further divided into familial PPNAD as a part of the Carney complex (CNC) (cPPNAD) and isolated PPNAD.

CNC is a rare genetic condition in which benign connective tissue tumors develop in the heart, skin, mammary gland, bone, and endocrine system, including the adrenal cortex, pituitary gland, thyroid gland, testicle, and ovary. PPNAD, which occurs in 25–60% of CNCs, is known as cPPNAD, is more frequent in women, and can appear sporadically in 20–30% of cases [85]. cPPNAD develops as a result of an inactivating mutation in the *PRKAR1A* gene, which encodes the PKA regulatory subunit type 1a and enhances PKA activity. In contrast, PPNAD, which is not connected to the CNC, is known as isolated PPNAD and accounts for approximately 10% of PPNAD [86]. They mostly cause hyperplasia, tumorigenesis, and steroidogenesis through mutations in the *PRKACA*, *PRKAR1A*, and *PDE11A* genes, with minor effects from *PDE8B* and *PRKACB* gene mutations [87,88,89,90,91,92]. Several mutations and the resulting molecular mechanisms that appear in various adrenal hyperplasia cases are summarized in Table 1.

### 3.2. Cortisol-Producing Adenoma

Cortisol-producing adenomas (CPA) cause overt Cushing’s syndrome with symptoms and subclinical Cushing’s disease without symptoms. Unlike adrenocortical hyperplasia, CPA voluntarily causes steroidogenesis; hence, therapy is performed via resection rather than prescription medicine. However, this was caused by a gene mutation similar to the gene mutations in adrenocortical hyperplasia described earlier [87,97,98,109,110]. Somatic mutations cause approximately 70% of CPA, with *PRKACA* being the most prevalent cause, accounting for 35–66% of CPA, with the majority occurring in the hotspot area of the gene, with a leucine residue at position 206 [110,111,112]. This mutation induces hypercortisol synthesis by disrupting the link between the catalytic and regulatory subunits of PKA, leading to cAMP-independent activity, CREB1 activation, and increased ferredoxin 1 expression via *FDX1* gene activity [81,111,113].

In contrast, the *GNAS* mutations are the most frequent in subclinical Cushing’s disease, accounting for approximately 70% of subclinical Cushing’s syndrome cases [114]. Eighty percent of *GNAS* mutations are missense, primarily affecting the serine residue at position 45 harbored in the consensus GSK3-β/CK1 phosphorylation site [114,115]. This alteration promotes Wnt/β-catenin signaling by preventing β-catenin phosphorylation.

Similarly, *CTNNB1* mutations are observed in 70% of adrenocortical adenomas; however, the majority are non-secreting adrenocortical adenomas (61%), with a few cases of subclinical Cushing’s disease (22%) and overt Cushing’s disease (16%). This mutation accounts for approximately 23% of the total CPA and stimulates the Wnt/β-catenin pathway. Wnt/β-catenin activation may be more strongly associated with tumorigenesis and aldosterone production by differentiation and maintenance of aldosterone-producing adrenal ZG, not cortisol production [106,116,117,118].

Additionally, minor somatic mutations in *PRKACB* p.S54L increase PKA activity, leading to tumorigenesis [109]. Furthermore, 23% of patients with CPA had a *PRKAR1A* gene inactivation mutation, which led to elevated cAMP and further Wnt/β-catenin signaling via PKA activation [106,119].

These gene mutations influence tumor development and cortisol production via the following mechanisms: increased *HSD3B1* mRNA levels in CPA have a significant impact on cortisol overproduction. At this point, NR5A1, GATA6, and NR4A1 play vital roles in regulating the transcription of *CYP11B1*, which is required for cortisol production [120].

Furthermore, locally exclusive cortisol secretion from CPA to the surrounding area involves intratumoral immune cell infiltration, angiogenic chemokines (chemokine (C-X-C motif) ligand (CXCL)1 and CXCL2), vascular density, p16, and p21, which are positive indicators that all CPAs affect the composition of CPA-specific microenvironments. Local cortisol secretion from the CPA leads to immune cell infiltration, increased angiogenic chemokines (CXCL1 and CXCL2), increased vascular density, abundant p16 and p21, and positive senescence-associated β-galactosidase, affecting the composition of the CPA-specific microenvironment [121]. In rare cases of CPA during pregnancy, DNA methylation changes influence the Wnt/β-catenin, Ras/MAPK, and PI3K/AKT pathways, resulting in accelerated tumor growth [122].

### 3.3. Primary Aldosteronism

Primary aldosteronism (PA) is distinguished by aldosterone synthesis, which is independent of the renin-angiotensin system. PA is caused by 60% idiopathic adrenal hyperplasia (IAH), 30% aldosterone-producing adenoma (APA), and the less common adenocarcinoma and familial hyperaldosteronism (FH) [123]. PA increases aldosterone levels, which causes various degrees of hypertension, hyperkalemia, and serious cardiovascular events [124].

#### 3.3.1. Idiopathic Adrenal Hyperplasia

Because only 5% of IAH cases are caused by a family background, it seems unlikely that the condition is hereditary [125]. The HISTALDO classification, which separates unilateral adrenocortical lesions with CYP11B2 IHC positive, was recently adopted [56]. This classification has the advantage of containing only functional aldosteronism because it is CYP11B2 positive and, histologically, it further subdivides the adrenocortical hyperplasia into aldosterone-producing nodules with a size of less than 1 cm, aldosterone-producing micronodules (APM) with invisible nodules, and a diffuse type of aldosterone-producing diffuse hyperplasia. As a recently suggested classification, research on associated genetic mutation is lacking; however, *CACNA1D* mutation is more common in aldosterone-producing nodules than other hyperplasia and tumors, and gathering such data is expected to help targeted therapy [126].

IAH involves at least one APM, also known as the CYP11B2-positive aldosterone-producing cell cluster, which is larger and more prevalent than normal cells. Furthermore, 58% of the somatic mutations in the *CACNA1D* gene, which encodes the L-type calcium voltage-gated channel subunit alpha 1-D in APM, were observed in IAH, indicating a strong link to this gene [127]. This mutation eventually increases the calcium influx via calcium channel activation, resulting in hyperaldosteronism [128]. Furthermore, a rare *CLCN2* mutation at c.197.G>A and c.143C>G causes aldosteronism by increasing the Cl current [129]. According to a recent mouse experiment, TASK channel deletion is comparable to real IAH because PA can occur regardless of renin; however, further research is needed to evaluate its clinical relevance [130].

#### 3.3.2. Aldosterone-Producing Adenoma

APA accounts for 30% of PA cases and is strongly related to *ATPA1A*, *ATP2B3*, *KCNJ5*, and *CACNA1D* gene mutations occurring in more than 50% of cases [131,132]. The *ATPA1A* gene mutation causes aberrant Na^+^/K^+^ ATPase α subunit, resulting in persistent cell depolarization, and the *ATP2B3* gene mutation causes abnormal Ca^2+^ ATPase, impairing cytoplasmic Ca^2+^ clearance and producing aldosterone syndrome [133]. Furthermore, 35–45% of APAs are caused by inactivating mutations in the *KCNJ5* gene, of which two hotspot somatic mutations, G151R and L168R, account for more than 40% [134]. Moreover, it is particularly common in young women and promotes excessive aldosterone synthesis through excessive sodium influx and increased expression of *CYP11B2* and *NR4A2* [135,136]. Additionally, in APAs with *KCNJ5* gene mutations, cholesterol ester uptake, and de novo cholesterol synthesis via SR-B1 increase, resulting in greater steroidogenic metabolism [137]. The *CACNA1D* mutation results in the expression of an aberrant L-type calcium channel that enhances calcium influx via calcium channel activation [128]. Particularly, 77% of the somatic mutations in the *CACNA1D* gene arise from APM-harboring APA [127]. Similarly, a rare *CACNA1H* mutation (in 4% of APAs) encodes an aberrant T-type calcium channel alpha-1H subunit that enhances calcium influx [138]. Moreover, *CLCN2* mutation (1%), an uncommon form, induces depolarization via chloride channel protein 2 [139,140]. Mutation of the *CLCN2* gene, which encodes a chloride channel, causes increased chloride permeability and depolarization in APA, eventually leading to aldosteronism via calcium influx [129]. Mutations in the *CTNNB1* gene lead to β-catenin accumulation, which increases *CYP11B2* expression and aldosterone synthesis [141]. Recently, it has been reported that LHCGF expression is further elevated by double mutation of *CTNNB1* and *GNA11/Q* (encoding G-protein subunit alpha 11), aggravating the aldosteronism of APA, which has been observed to be associated with pregnancy, menopause, or puberty [142]. *GNAS* and *ARMC5* mutations are strongly associated with CPA or PBMAH; however, APA has been reported [70,143]. Additionally, a recent study reported that a somatic mutation in the *SLC30A1* gene, which encodes the zinc efflux transporter ZnT1, acts on His43 and Asp47, the zinc-binding sites in transmembrane domain II, producing aberrant ion transport [144]. The resulting aberrant Na^+^ conductance causes depolarization of the cell membrane and cytosolic Ca^2+^ activity, thereby boosting aldosterone synthesis. This mutation increases the levels of numerous cytokines in the bloodstream, including IP-10, CXCL9, and RANTES, further boosting blood pressure and heart rate [145]. Recently, p.Val380Asp and p.Gly379Asp variants of *CADM1* were reported in APA, and the mutation or suppression of that gene raises CYP11B2 of ZG, inducing aldosteronism [146]. According to a recent bioformatics study, MEG3 and LINC00115 are the principal regulators of lncRNAs, and 9 miRNAs and 13 mRNAs have a significant impact, necessitating further genetic experiments [147].

#### 3.3.3. Familial Hyperaldosteronism

FH is extremely rare in comparison to other forms of PA and can be identified when an individual has PA and at least one first-degree relative is affected. FH is classified into four categories based on the type of germline mutation, and the level of aldosteronism varies according to the variant. FH-I is a PA known as glucocorticoid-remediable aldosteronism, or glucocorticoid-suppressible hyperaldosteronism, and is inhibited by dexamethasone. This is caused by the hybridization of the *CYP11B1* and *CYP11B2* genes via asymmetric crossover, and the *CYP11B1*/*CYP11B2* fusion enzyme is expressed and activated under the control of ACTH rather than angiotensin II and potassium [148]. According to the most recent study, compared with other types of FH, it arises at a young age, is more common in women, and has a weaker degree of aldosteronism (lower plasma aldosterone concentration, higher plasma renin activity, and less frequent hypokalemia) [149]. This is probably due to the mechanism controlled by ACTH. In contrast, all other FHs are caused by germline mutations without a hybrid gene, and dexamethasone does not prevent aldosterone overproduction. Recent research has established that FH-II, the most frequent type of FH, has a germline mutation of the *CLCN2* gene in common, which is produced by variants such as p.Arg172Gln, p.Met22Lys, p.Tyr26Asn, p.Lys362del, and Ser865Arg [150]. FH-III is caused by a germline mutation in the *KCNJ5* gene, which causes aberrant K^+^ loss, increased Na^+^ influx into the cytoplasm, increased intracellular Ca^2+^ levels, and depolarization, leading to aldosteronism [151]. FH-III had a greater phenotype than the other FHs, with higher plasma aldosterone concentrations and a greater frequency of hypokalemia [149]. FH-IV encodes an aberrant calcium channel unit through a germline mutation in the *CACNA1H* gene, increasing Ca^2+^ influx and the activation of *CYP11B2* and other steroidogenic genes [152]. Moreover, although FH has not yet been recognized, germline mutations in *CACNA1D* (also known as PA with seizures and neurological abnormalities) and inherited aldosteronism via the *ARMC5* gene have been reported [128]. A more detailed classification of FH will assist in future targeted treatments.

### 3.4. Adrenocortical Carcinoma

Adrenocortical carcinoma (ACC) is a rare but highly aggressive malignant tumor, with a 5-year survival rate of 35%. In contrast to adenomas, functioning ACC secretes androgen, cortisol, estrogen, and aldosterone at rates of 85% in children and 30% in adults [153]. Furthermore, ACC has reduced levels of the essential steroidogenic enzymes CYP11B1, CYP11B2, and CYP17A1, showing an incomplete steroidogenesis pattern compared with adenoma [154].

Although the mechanisms of carcinogenesis in ACC are not entirely understood, the most deeply associated signaling and somatic mutations in ACC are *TP53* and *IGF-2* gene alterations, as well as Wnt/β-catenin signaling, which are closely related to cell proliferation [155,156,157]. ACC is associated with somatic mutations in *CTNNB1*, *APC*, *MEN1*, *BRCA1*, *BRCA2*, and *PRKAR1A*, similar to those in adrenocortical adenoma or hyperplasia, as well as in *NF1*, *NF2*, *RB1*, *CDC27*, *SCN7A*, *SDK1*, *TERT*, *ZNRF3*, *ATRX*, *NOTCH1*, *CIC*, *KDM6A*, and *RPL22* genes [158,159,160]. Furthermore, patients with ACC have 2–53 times greater expression of *TOP2A*, *IGF2*, *CDK1*, *CDK4*, *PLK4*, and *PLK1* genes than normal controls, of which the overexpression of *CDK4* causes tumorigenesis via p53/Rb1-pathway-related cell cycle alterations [161]. MSH2 loss caused by *TP53* mutation and cortisol hypersecretion caused by *CTNNB1* mutations were identified in a metastatic cancer cell line with aldosterone-secreting ACC and Lynch syndrome [162]. These mutations occur 2.8 times more frequently in metastatic ACC than in primary ACC [163]. ACC is associated with germline mutations. Particularly, 50% of pediatric patients with ACC have a germline mutation in *TP53*, which is associated with an elevated risk of various primary malignancies [164].

Because ACC is a malignant tumor, many genetic mutations occur, and the factors related to ACC are still not well known. To reveal these various factors, in silico and bioinformatic analyses have been performed recently. According to in silico data, genes related to poor prognosis and recurrence, such as *ZWINT*, *PRC1*, *CDKN3*, *CDK1*, and *CCNA2*, are differentially expressed in ACC [165]. Additionally, bioinformatics analysis revealed that different N6-methyladenosine (m6A) modification patterns alter the tumor immune microenvironment, and *HNRNPA2B1*, an m6A regulator, is associated with poor prognosis and ACC progression [166]. Furthermore, *ANLN*, *ASPM*, *CDCA5*, *CENPF*, *FOXM1*, *KIAA0101*, *MELK*, *NDC80*, *PRC1*, *RACGAP1*, *SPAG5*, and *TPX2*, which play important roles in tumor progression, metastasis, DNA damage repair, and hematopoiesis, are associated with poor outcomes and tumor stage [167].

Cancer stem cells are a small subset of cancer cells that possess properties similar to normal stem cells [168]. As a result, cancer stem cells have an enhanced ability to initiate tumor growth, proliferation, invasion, and migration while also resisting therapeutic interventions. Although adrenocortical stem cells have a limited steroidogenic capacity, making them less directly relevant to the treatment of adrenocortical hyperfunction, inhibiting cancer stem cells and restricting metastasis and differentiation could still be highly beneficial in managing adrenocortical hyperfunction [169]. Using bioinformatics methods, ACC patients with a high mRNA stemness index, which reflects the transcriptomic stemness features, showed shorter overall survival and a higher metastatic tendency [170]. Furthermore, lower expression of PD-L1, CTLA-4, and TIGHT was observed in ACC patients with a high mRNA stemness index. This information could be useful in evaluating the effectiveness of immunotherapy and in designing targeted therapeutic strategies.

Recent studies have focused on miRNA and lncRNA alterations in cancers and, as a result, therapeutic approaches have been developed. ACC is known to downregulate *miR-19* (decreased apoptosis) and *miR-7* (enhanced cell viability and invasiveness) while upregulating *miR-483* (increased *IGF2* expression and regulation of the BBC3/PUMA protein) [171,172,173]. Overexpression of *miR-503* and *miR-210* and downregulation of *miR-195* and *miR-335* alter apoptosis and cell proliferation, leading to poor prognosis [174]. Furthermore, reduced expression of lncRNA *PRINS* is related to ACC recurrence and metastasis [175]. Recently, changes in extracellular-vesicle-associated miRNAs in patient plasma have been confirmed, allowing for a convenient and rapid diagnosis. Higher expression of miRNAs, including *miR-101* and *miR-483-5p*, has been detected in patients with ACC than in those with adrenocortical adenoma, suggesting that these miRNAs are useful diagnostic and prognostic markers [176].

### 3.5. Androgen-Secreting Adrenal Tumor

Hyperandrogenism is a condition in which the body produces an excessive amount of androgen, resulting in acne, hirsutism, alopecia, menstrual dysfunction, and infertility. It is more common in women, accounting for 5–10% of the population. However, the majority of adrenal-disease-induced hyperandrogenism is secondary hyperandrogenism caused by congenital adrenal hyperplasia [177]. Furthermore, androgen-secreting adrenal tumor (ASAT) occurs in one to two cases per million people each year, and hyperandrogenism occurs in 0.1% of adrenocortical tumors, which is extremely rare [178,179,180]. Most androgen-secreting adrenal tumors are malignant and do not respond to dexamethasone [181]. A study reported three positive cases against p53 and two positive cases against β-catenin in eight ASATs [182]. Additionally, two patients had mutations in the *AIP* gene (p.Lys177Argfs19 and ASP28Val). In addition, one single ASAT patient had elevated *CYP11A1*, *CYP17A1*, *CYB5A*, and *AKR1C3*, as well as decreased *HSD3B1*, *HSD3B2*, and *CYP21A2* levels [183]. However, the tumor is extremely rare and there is currently not enough data; therefore, additional research is required.

## 4. Treatment

**Figure 5:**
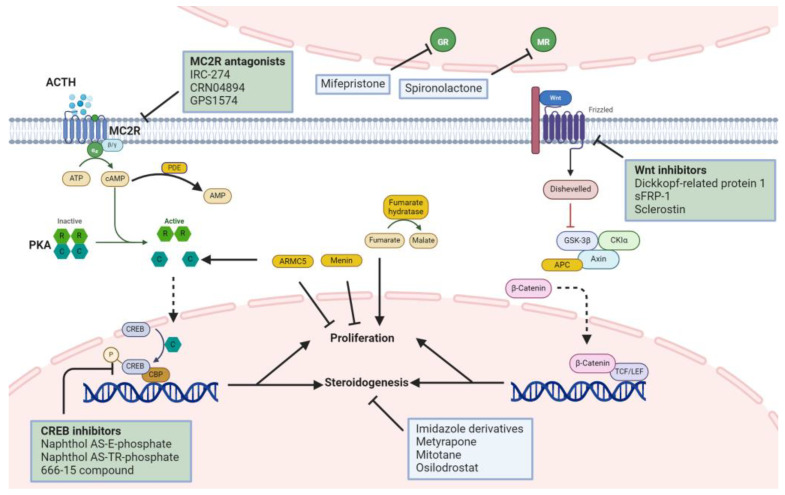
Diagram of conventional and anticipated drugs for adrenal hyperfunction. Blue boxes indicate conventional drugs and green boxes indicate anticipated drugs for adrenal hyperfunction. Straight solid arrows (→) indicate activation or a direct progression of a process; dashed arrows (---→) indicate an anticipated outcome; inhibition arrows (⊥) indicate inhibition or suppression of a pathway or protein activity. Abbreviations: ACTH, adrenocorticotropic hormone; MC2R, melanocortin type 2 receptor; cAMP, cyclic adenosine monophosphate; PDE, phosphodiesterase; PKA, protein kinase A; *PRKACA*, protein kinase A catalytic subunit α; *PRKAR1A*, protein kinase A regulatory subunit α; *PRKACB*, protein kinase A catalytic subunit β; *ARMC5*, armadillo repeat containing 5; *MEN1*, multiple endocrine neoplasia type 1; *CTNNB1*, catenin beta 1; *APC*, adenomatous polyposis coli; CREB, cAMP-response element binding protein; CBP, CREB-binding protein; GR, glucocorticoid receptor; MR, mineralocorticoid receptor; sFRP-1, secreted Frizzled-related protein 1.

Adrenal resection is extensively used to treat adrenocortical hyperfunction; however, it may result in persistent insufficiency and be ineffective in patients with metastases or difficult surgeries. Radiation therapy is quite successful in controlling adrenocortical tumors, and the remission rate is high, approximately 83–100% [184]. However, endocrine remission is longer and the duration varies depending on the indication. As a result, various pharmaceuticals are being used and attempts are underway to develop novel therapies for targeted treatment based on molecular mechanisms (Figure 5).

### 4.1. Conventional Treatment of Adrenocortical Hyperfunction

Steroidogenesis inhibitors suppress the production of steroids regardless of the etiology of adrenocortical hyperfunction and can control approximately 70% of adrenocortical hyperfunctions [185]. Ketoconazole is an imidazole derivative that inhibits CYP11A1, CYP11B1, and CYP17 and inactivates ACTH secretion to inhibit steroidogenesis [186]. However, because of adverse effects, such as nausea, dyspepsia, and hepatotoxicity, they are used with caution [185,187]. Etomidate, also an imidazole derivative, inhibits cortisol production by inhibiting CYP11A1, CYP11B1, and CYP17; however, excessive sedation should be noted because its original use was anesthesia [188,189]. Metyrapone is an adrenal steroid synthesis inhibitor that inhibits cortisol synthesis primarily through CYP11B1, with modest inhibitions of CYP17, CYP11B2, and CYP19 [185]. Metyrapone efficiently manages glycemia, hypertension, and hypokalemia in 50–70% of patients with Cushing’s syndrome [187,190]. However, it can lead to the accumulation of mineralocorticoids and androgen precursors, resulting in hypertension and hirsutism [191]. Mitotane is a derivative of dichlorodiphenyltrichloroethane that inhibits CYP11B1, CYP11B2, and 3β-HDS. Moreover, it causes lipid accumulation and adrenolysis at high concentrations [185]. Although the remission rate can reach 72% after six months of treatment, it should be used with caution because of various adverse effects, including gastrointestinal and neurological issues [185,192]. Mifepristone is a GR and PR antagonist that inhibits the activity of glucocorticoids and lowers glycemia and body weight in patients with Cushing’s syndrome [193]. Additionally, excessive use might lead to hypoadrenalism and should be proceeded with caution. Osilodrostat, a medication approved by the European Medicines Agency and U.S. Food and Drug Administration in 2020, reduces cortisol production by inhibiting CYP11B1 [194]. Additionally, it plays a role in PA by inhibiting aldosterone synthase [195].

The primary goal of aldosteronism treatment is to control hypertension and hypokalemia. To reduce blood pressure, several medications are utilized, including diuretics, angiotensin I converting enzyme inhibitors, beta-blockers, α2-adrenergic agonists, calcium channel blockers, and renin inhibitors [196]. However, diuretics can exacerbate hypokalemia, and certain blood-pressure-lowering medications should be used with caution because of the potential reduction in blood pressure, renal filtration, and glomerular filtration rate [197,198]. However, unlike other diuretics, spironolactone prevents excessive potassium loss by antagonizing the aldosterone receptor. Combination therapy is widely used to treat adrenocortical hyperfunction rapidly while lowering adverse pharmacological effects and it has shown higher success rates.

### 4.2. Anticipated Drugs Related to the Molecular Mechanism

Various medications inhibit the mechanisms that induce adrenocortical hyperfunction; however, many have not yet been used on the adrenal gland. The primary reason is assumed to be the greater adverse effects rather than the regulating role of adrenocortical hyperfunction of the related pathways. The application of the following medications may assist in better-targeted treatment with minimal side effects.

#### 4.2.1. MC2R Receptor Antagonist

IRC-274, an MC2R antagonist, reduces circulating corticosterone levels by inhibiting MC2R in HEK 293 cells and rat models [199]. IRC-274 is expected to have few adverse effects because it specifically works on the MC2R and does not affect other types of melanocortin receptors (MC1R, MC3R, MC4R, and MC5R) involved in the immune response [200]. CRN04894 is also an MC2R selective antagonist that acts as a competitive antagonist of ACTH, suppressing basic cortisol and ACTH-stimulated corticosterone production [201]. Similarly, GPS1573 and GPS1574 antagonize MC2R in vitro and have extremely low potency against melanocortin receptors [202]. However, as only a high dose of GPS1574 was observed to antagonize MC2R in vivo testing, only this medicine is predicted to be used as a treatment for adrenal hyperfunction [203]. Because these medications, ironically, inhibit ACTH, they are expected to replace some of the risks and side effects associated with steroid treatments used to antagonize excessive ACTH in congenital adrenal hyperplasia caused by autosomal recessive adrenal dysfunction [204].

#### 4.2.2. Wnt Inhibitor

Wnt inhibitors reduce cytoplasmic β-catenin accumulation, thereby suppressing tumorigenesis. Dickkopf-related protein 1, a typical Wnt inhibitor, has a negative feedback effect on human osteoblasts via steroids [205]. Although not directly applied to adrenal tumors, it is known to be effective against gastrostatic tumors [206]. However, because of its ability to evade immune elimination by dendritic cells, T cells, and natural killer cells, Dickkopf-related protein 1 should be used with caution [207,208,209]. Secreted Frizzled-related protein 1 (sFRP-1) is secreted from osteoblasts and bone marrow cells in response to excess glucocorticoids [210]. In addition, silencing of sFRP-1 plays a major role in the survival of clear cell renal cell carcinoma and triple-negative breast cancer tumor cells, and the addition of recombinant sFRP-1 triggers apoptosis in clear cell renal cell carcinoma and triple-negative breast cancer tumor cells to restrict cell growth [211]. Sclerostin is an osteocyte-secreted molecule that inhibits Wnt signaling. The proliferation, migration, and cell survival of osteosarcoma cells is inhibited in mice and humans, whereas sclerostin blockade stimulates canonical Wnt signaling to promote metastasis in breast carcinoma cells [212,213]. Tegavivint is a small molecule that inhibits the Wnt/β-catenin axis, preventing the mouse ACC model’s cells from growing both in vivo and in vitro [214]. Nevertheless, liver and kidney stress as well as off-target effects should be considered when using Wnt inhibitors.

#### 4.2.3. CREB Inhibitors

CREB is important for tumor development. CREB indicates a poor prognosis for many malignancies, including melanoma, pancreatic tumors, breast cancer, gliomas, and non-small cell lung cancer [215,216,217,218,219]. Typical CREB inhibitors include the CREB-binding protein (CBP) and cAMP-response element (CRE) DNA inhibitors. CBP binds to CREB, which is phosphorylated by PKA, to initiate gene transcription [220]. Several CBP inhibitors, such as naphthol AS-E-phosphate, naphthol AS-TR-phosphate, and 666-15 compound, have demonstrated antitumor activity against various cancers, including leukemia, lung cancer, and breast cancer [221,222,223]. However, certain CBP inhibitors may cause hyperglycemia, which may worsen hyperglycemia, which is frequently observed in Cushing’s syndrome [224]. CRE is a DNA sequence that functions as a transcription promoter by binding to phosphorylated CREB and combining with it to create downstream substances [225]. Surfen and stibavirin are CRE DNA inhibitors that have shown antitumor effects against glioblastoma [226]. However, these medications have not yet been tested in adrenal tumors; therefore, further research is required.

## 5. Conclusions

The patient’s quality of life is greatly reduced by adrenal hormonal imbalance because the adrenal gland is an organ that controls hormones in multiple organs. It has been known that people with adrenal hyperfunction and hypofunction have a very low quality of life, and the mortality rate is also high, with a median survival of 4.6 years; therefore, treating adrenal gland disease is critical [227,228]. In treating primary adrenocortical hyperfunction, managing only increased hormones is not appropriate as a fundamental treatment. Adrenalectomy, the most often used treatment, has been linked to a high proportion of postoperative complications, death, and adrenal crisis caused by sudden AI, and various medications also have been reported as having side effects [229].

Recent advances in understanding the molecular mechanisms underlying adrenocortical hyperfunction disorders, particularly genetic mutations in key pathways, offer promising avenues for more personalized and targeted therapies. Genetic mutations predominantly present in each primary adrenal hyperfunction, such as *ARMC5* (hypercortisolism), *CACNA1D* (aldosteronism), and *AIP* (hyperandrogenism), disrupt key molecular pathways, including the cAMP/PKA/CREB signaling pathway and Wnt/β-catenin signaling, leading to uncontrolled hormone synthesis and tumorigenesis. In addition to the previously described anticipated drug targets, understanding the precise molecular mechanisms involved in these pathways provides critical insight for the development of novel therapeutic approaches. Furthermore, a new and more precise classification that incorporates these molecular pathways and histological characteristics would pave the way not only for advanced targeted therapy but also for detailed clinical staging and specific decisions. We hope that this review will provide information on the molecular and genetic mechanisms of the most recent primary adrenocortical hyperfunction, offer clinicians a variety of treatment options, and help researchers with guidance for associated tumor studies.

## Figures and Tables

**Table 1:** Adrenal hyperplasia and related mutations.

Adrenal Hyperplasia	Associated Mutated Gene	Associated Locus	Function of the Mutated Gene	Reference
PBMAH	*ARMC5*	16p11.2	Decreased apoptosis Increased tumorigenesis Preventing interaction with Culin3 Inhibiting MC2R expression Reduced *CYP17A1* and *CYP21A2* mRNAs Inhibiting cAMP/PKA/CREB signaling Disrupted redox homeostasis Inhibiting/increasing steroidogenesis Aberrant expression of β-adrenergic, α2-adrenergic, and V1-vasopressin receptors Steroidogenesis by inactivating SIRT1	[60,62,63,64,65,66,67,68,69]
	*KDM1A*	1p36.12	GIP-receptor overexpression Promoting GIP/cAMP/PKA/CREB signaling Cell proliferation and steroidogenesis	[72,93]
	*GIPR*	19q13.32		
	*MEN1*	11q13	Decreased menin encoding Tumorigenesis by menin repression Inhibiting cAMP/PKA/CREB signaling	[77,94,95]
	*Fumarate hydratase*	1q42.3-43	Decreased malate formation from fumarate Tumorigenesis by fumarate Inhibiting cAMP/PKA/CREB signaling	[77]
	*PDE11A*	2q31-35	cAMP hydrolysis Inhibiting cAMP/PKA/CREB signaling	[96,97]
	*PDE8B*	5q13	cAMP hydrolysis Inhibiting cAMP/PKA//CREB signaling	[98]
	*GNAS*	20q13	Increased G-protein alpha subunit encoding Promoting GNAS/cAMP/PKA/CREB signaling	[74]
	*APC*	5q21-22	Promoting Wnt/β-catenin signaling Inhibiting cAMP/PKA/CREB signaling	[77,99]
	*MC2R*	18p11	Activating MC2R Promoting MC2R/cAMP/PKA/CREB signaling	[82,83]
	*PRKACA*	19p13.1	Inactivate negative regulatory subunit RIIβ binding site Promoting PKA/CREB signaling	[87]
	*DOT1L*	19p13.3	Deregulating cell proliferation	[81]
	*HDAC9*	7p21.1	-	[81]
	*GPC4*	Xq26.2	Connected to cancer-related pathway, Rap1 signaling pathway, PI3K-AKT signaling pathway, Wnt signaling pathway	[84]
	*VCAN*	5q12-14	Connected to PI3K-AKT signaling pathway, phospholipase D signaling pathway, Rap1 signaling, Ras signaling pathway, MAPK signaling pathway	[84]
cPPNAD	*PRKAR1A*	17q24.2 2p16	Decreased regulatory subunit of PKA (Haploinsufficiency) formation Increased cAMP/PKA/CREB signaling	[100,101,102]
	*PDE11A*	2q31-35	cAMP hydrolysis Inhibiting cAMP/PKA/CREB signaling	[103]
	*PRKACB*	1p31.1	Increased protein kinase catalytic subunit β Increased cAMP/PKA/CREB signaling	[104]
i-PPNAD	*PRKACA*	19p13.1	Inactivate negative regulatory subunit RIIβ binding site Promoting PKA/CREB signaling	[87]
	*PRKAR1A*	17q24.2	Decreased regulatory subunit of PKA (Haploinsufficiency) formation Increased cAMP/PKA/CREB signaling	[88,89]
	*PDE11A*	2q31-35	cAMP hydrolysis Inhibiting cAMP/PKA/CREB signaling	[90]
	*PDE8B*	5q13	cAMP hydrolysis Inhibiting cAMP/PKA/CREB signaling	[91]
	*PRKACB*	1p31.1	Increased protein kinase catalytic subunit β Increased cAMP/PKA/CREB signaling	[92]
	*CTNNB1*	3p22.1	Increased Wnt/β-catenin pathway	[105,106]
i-MAD	*PDE11A*	2q31-35	cAMP hydrolysis Inhibiting cAMP/PKA/CREB signaling	[90]
	*PDE8B*	5q13	cAMP hydrolysis Inhibiting cAMP/PKA/CREB signaling	[91]
	*PRKACA*	19p13.1	Inactivate negative regulatory subunit RIIβ binding site Promoting PKA/CREB signaling	[107]
	*PRKAR1B*	7p22	Increased regulatory subunit β of PKA Decreased cAMP/PKA/CREB signaling	[108]

Abbreviations: PBMAH, primary bilateral macronodular adrenal hyperplasia; *ARMC5*, armadillo repeat containing 5; MC2R, melanocortin type 2 receptor; cAMP, cyclic adenosine monophosphate; PKA, protein kinase A; *KDM1A*, lysine (K)-specific demethylase 1A; *GIPR*, glucose-dependent insulinotropic polypeptide receptor; *MEN1*, multiple endocrine neoplasia type 1; *PDE11A*, phosphodiesterase 11A; *PDE8B*, phosphodiesterase 8B; *GNAS*, guanine nucleotide-binding protein alpha stimulating activity polypeptide; *APC*, adenomatous polyposis coli; PRKACA, protein kinase A catalytic subunit α; cPPNAD, Carney complex primary pigmented micronodular adrenocortical disease; i-PPNAD, isolated primary pigmented micronodular adrenocortical disease; i-MAD, isolated micronodular adrenocortical disease.

## Abbreviations

ZG	Zona glomerulosa
ZF	Zona fasciculata
ZR	Zona reticularis
AI	Adrenal insufficiency
ACTH	Adrenocorticotropic hormone
GR	Glucocorticoid receptor
MR	Mineralocorticoid receptor
AR	Androgen receptor
3β-HSD	3β-hydroxysteroid dehydrogenase
DHEA	Dehydroepiandrosterone
DHEAS	Dehydroepiandrosterone sulfate
HPA	Hypothalamus-pituitary-adrenal
CRH	Corticotropin-releasing hormone
cAMP	Cyclic adenosine monophosphate
MC2R	Melanocortin type 2 receptor
ER	Estrogen receptors
MAPK	Mitogen-activated protein kinase
PBMAH	Primary bilateral macronodular adrenal hyperplasia
ARMC5	Armadillo repeat containing 5
CREB	cAMP-response element binding protein
PKA	Protein kinase A
GPCR	G protein-coupled receptor
KDM1A	Lysine (K)-specific demethylase 1A
GIPR	Glucose-dependent insulinotropic polypeptide receptor
LH	Luteinizing hormone
PPNAD	Primary pigmented micronodular adrenocortical disease
CNC	Carney complex
cPPNAD	CNC PPNAD
CPA	Cortisol-producing adenoma
PA	Primary aldosteronism
IAH	Idiopathic adrenal hyperplasia
APA	Aldosterone-producing adenoma
FH	Familial hyperaldosteronism
APM	Aldosterone-producing micronodule
APCC	Aldosterone-producing cell cluster
ACC	Adrenocortical carcinoma
m6A	N6-methyladenosine
ASAT	Androgen-secreting adrenal tumor
sFRP-1	Secreted Frizzled-related protein 1
CBP	CREB-binding protein
CRE	cAMP response element
